# Recent Theories and Applications in Approximation Theory

**DOI:** 10.1155/2015/598279

**Published:** 2015-04-08

**Authors:** Fazlollah Soleymani, Predrag S. Stanimirović, Juan R. Torregrosa, Hassan Saberi Nik, Emran Tohidi

**Affiliations:** ^1^Department of Mathematics, Islamic Azad University, Zahedan Branch, Zahedan, Iran; ^2^Faculty of Sciences and Mathematics, University of Niš, Visegradska 33, 18000 Niš, Serbia; ^3^Instituto de Matemáticas Multidisciplinar, Universitat Politècnica de València, 46022 Valencia, Spain; ^4^Department of Applied Mathematics, School of Mathematical Sciences, Ferdowsi University of Mashhad, Mashhad, Iran; ^5^Department of Mathematics, Kosar University of Bojnord, P. O. Box 9415615458, Bojnord, Iran

Approximation theory is a deep theoretical study of methods that use numerical approximation for the problems of mathematical analysis. In practical use, it is typically the application of computer simulation and other forms of computation to problems in various scientific disciplines. Recently, numerical algorithms in approximation theory have been a major thrust of research with numerous applications.

This special issue was opened in the middle of 2014 and closed in February of 2015. A number of selected submissions were accepted for publication after strict reviews, which furnished significant improvements in the topics of the special issue and its related applications. The guest editors of this special issue hope that the published results could provide outstanding viewpoints for further studies.

The fundamental aim of this special issue was to provide new trends in the field of approximation theory and related applications in mathematics. The authors were invited to submit original research articles to stimulate the continuing efforts in numerical approximation of mathematical problems and related theories. The special issue provided a forum for researchers and writers to communicate their state-of-the-art improvements and to propose their new findings on approximation theory.

The topics of the accepted papers cover the area from theory to real applications. Some new schemes and their corresponding convergence analysis have been discussed for some numerical problems. Furthermore, they have been equipped with several numerical tests with some applications.

Now, we have the pleasure to present, the selected papers for this special issue as follows.

S. S. Motsa et al. presented a novel scheme for solving higher order nonlinear evolution partial differential equations (NPDEs). Their discussed approach combines quasi-linearisation, the Chebyshev spectral collocation method, and bivariate Lagrange interpolation. They also showed that there is congruence between the numerical results and the exact solutions to a high order of accuracy.

The paper of M. Sharifi et al. presented interesting iterative methods including three steps for solving nonlinear equations. Their iterative approach possesses eighth-order of convergence which is optimal in the sense of Kung-Traub while it is also derivative-free. An integral equation has also been solved as an application-oriented experiment.

T. Lotfi et al., in their paper, investigate an optimal three-step method which has eighth-order of convergence. Then, they applied a self-accelerator parameter with Newtonian interpolation using the highest possible degree to improve the R-order of convergence as much as possible, that is, from 8 to 12 without any additional functional evaluations. This meant that a high computational efficiency index has been obtained for solving nonlinear equations.

H. S. Nik and P. Rebelo presented an application of pseudospectral method for solving the hyperchaotic complex systems. The proposed method, called the Multistage Spectral Relaxation Method (MSRM), was based on a technique of extending Gauss-Seidel type relaxation ideas to systems of nonlinear differential equations, while using the Chebyshev pseudospectral methods to solve the resulting system on a sequence of multiple intervals. Finally, it has been used to solve famous hyperchaotic complex systems such as hyperchaotic complex Lorenz system and the complex permanent magnet synchronous motor.

Y. Zhao et al. in their paper proposed a semilocal convergence theorem for the inverse-free Jarratt method under new Holder conditions. In fact, a new error estimate has been attained. Finally, three examples were provided to show the application of their discussed theorem for numerical problem of solving nonlinear equations.

In the paper of R. Behl and S. S. Motsa, authors proposed some geometric variation of the fourth-order Ostrowski's method so as to obtain methods with eighth-order of convergence and four functional evaluations. In this way, their method is optimal in the sense of Kung-Traub and would be useful for a class of problems in approximation theory.

The authors in the paper titled “On a Derivative-Free Variant of King's Family with Memory” proposed derivative-free variants of the well-known King's family of methods for nonlinear equations. They designed the approximation of derivatives to be as accurate as possible so as to keep the rate of convergence four using the same number of function evaluations. Finally, an extension of the family as a method with memory possessing higher computational efficiency index has been attained.

The paper titled “On a Cubically Convergent Iterative Method for Matrix Sign” proposed an interesting interlink between solvers for nonlinear equations and their applications for computing matrix functions. In this paper, it was shown that the new scheme has global behavior with cubical rate of convergence. Finally, several examples were also included to show the applicability and efficiency of the proposed scheme and its reciprocal.

K. Muzhinji et al. presented the application of different smoothers and compared their effects in the overall performance of the multigrid solver. They studied the multigrid method with the following smoothers: distributed Gauss-Seidel, inexact Uzawa, preconditioned MINRES, and Braess-Sarazin type smoothers. Lastly, numerical results have been included to demonstrate the efficiency and robustness of the multigrid method and confirm the theoretical results.

Finally, B. Alkahtani presented the Homotopy Analysis Method (HAM) to obtain the analytical solutions of the general space-time fractional diffusion equation. The explicit solutions of the equations have been presented in the closed form by using initial conditions. Several examples were also discussed to confirm the method proposed in this paper.

